# Detection of Sound Image Movement During Horizontal Head Rotation

**DOI:** 10.1177/2041669516669614

**Published:** 2016-09-16

**Authors:** Akio Honda, Kagesho Ohba, Yukio Iwaya, Yôiti Suzuki

**Affiliations:** Yamanashi Eiwa College, Yamanashi, Japan; Tohoku University, Sendai, Japan; Tohoku Gakuin University, Miyagi, Japan; Tohoku University, Sendai, Japan

**Keywords:** sound localization, virtual sound source, active listening, rotation velocity

## Abstract

Movement detection for a virtual sound source was measured during the listener’s horizontal head rotation. Listeners were instructed to do head rotation at a given speed. A trial consisted of two intervals. During an interval, a virtual sound source was presented 60° to the right or left of the listener, who was instructed to rotate the head to face the sound image position. Then in one of a pair of intervals, the sound position was moved slightly in the middle of the rotation. Listeners were asked to judge the interval in a trial during which the sound stimuli moved. Results suggest that detection thresholds are higher when listeners do head rotation. Moreover, this effect was found to be independent of the rotation velocity.

## Introduction

For azimuthal sound localization, we use the interaural time difference and the interaural level difference (Blauert, 1997). This information changes according to the listener’s head movement, thereby influencing sound localization accuracy. Wallach (1939) demonstrated that this information provides clues for the elevation angle judgment at sound image localization and for front or back judgment. Similar findings were obtained using a virtual reality system. For instance, Kawaura, Suzuki, Asano, and Sone (1989) investigated effects of head movement on sound image localization using a 3D virtual auditory display (VAD). They reported that front or back judgment of the sound localization task is improved by reflecting a listener’s head movement. As described herein, many reports have described that sound localization is facilitated by head movement, which creates dynamic changes to the information input to both ears.

It is particularly interesting that recent reports have described that these effects differ depending on the sound features (Iwaya, Suzuki, & Kimura, 2003; Perrett & Noble, 1997), and that the sound image localization accuracy is reduced by head movement depending on the timing of the sound presentation (Cooper, Carlile, & Alais, 2008; Leung, Alais, & Carlile, 2008). For instance, Iwaya et al. (2003) revealed that the listener’s head rotation reduces the front–back error effectively when the sound duration is long. Moreover, Cooper et al. (2008) performed a task by which a test sound was presented while the listener was engaged in the head rotation movement, and the listener was asked to report the sound image position when the rotation movement was finished. They reported the result that presentation of sound stimulation during head rotation movement caused reduction in the sound image localization accuracy compared with a case in which the head is not moved. Leung et al. (2008) examined auditory spatial perception during rapid head motion. They demonstrated that the perception of auditory space was compressed during rapid head motion for stimuli occurring in the perisaccadic interval. The authors’ group assessed whether listener’s horizontal head rotation affects sound localization accuracy at the subjective front (Masumi, Honda, Suzuki, & Sakamoto, 2014; Masumi, Suzuki, Honda, & Sakamoto, 2014). Results showed that reduction in sound localization accuracy during head rotation movement occurred with passive rotation movement (Masumi, Honda, et al., 2014) and active rotation movement (Masumi, Suzuki, et al., 2014). Recently, Brimijoin and Akeroyd (2014) reported that the moving minimum audible angle for virtual sound sources during self-motion is larger than in the still condition but is smaller than that during sound source motion. Nevertheless, few researchers have examined sound source localization when listeners and sounds rotate (Yost et al., 2015). Therefore, this study examined sound image movement detection during horizontal head rotation using the VAD.

## Experiment 1

Observers (listeners) were eight male students with normal hearing ability (average age: 22.9 years old). To present a sound source with arbitrary movement with no mechanical noise, it was presented virtually via a VAD, which outputs binaural output signals via the headphones after convolving head-related impulse responses (HRIRs) of a specified sound source position. Actually, HRIRs are the time domain representation of head-related transfer function (Blauert, 1997).

This VAD was developed by the authors’ group (Yairi, Iwaya, & Suzuki, 2007, 2008). The system consisted of a pair of headphones, a magnetic position sensor, and a personal computer (3.06 GHz Pentium 4 CPU, 2 Gbyte memory). The operating system of the personal computer was Linux (kernel 2.6). The sound driver was Open Sound System with a sound card (Audiophile 2496; M-Audio). Actually, HRIRs (head-related transfer functions) are determined according to anthropometrical parameters such as the sizes and shapes of the listener’s ears, head, and body. They are, therefore, strongly idiosyncratic, differing among individuals (Morimoto & Ando, 1980; Watanabe, Iwaya, Suzuki, Takane, & Sato, 2014; Watanabe, Ozawa, Iwaya, Suzuki, & Aso, 2007). Therefore, the use of individual HRIRs (individualization) is extremely important to achieve good sound localization with VADs (Morimoto & Ando, 1980). To do this, HRIRs were measured for all listeners with a spherical speaker array installed in an anechoic room at the authors’ institute. HRIRs for sound sources located 1.5 m from the center of the spherical array, where the center of listener’s head is aligned during HRIR measurements, were measured using an equal interval angle of 5° for the 17 horizontal planes from (80° to 80° for every 10° in the elevation angle, as well as the two poles. The sampling frequency was 48 kHz. To achieve smooth rendering, measured HRIRs were spatially interpolated for any direction with resolution of 0.1° based on an average with weights depending on the angle separation between the direction and the four directions around it where the measured HRIRs are available (Watanabe, Takane, & Suzuki, 2005; Yairi et al., 2007, 2008). Nishino, Kajita, Takeda, & Itakura (1999) evaluated the performance of the linear interpolation of HRIR using an objective measure based on spectral distortion and by a subjective listening test to show that sufficient precision in terms of sound localization is obtained if the spatial resolution of the measured HRIR is as small as 10°. For the four HRIR used for the linear interpolation, temporal alignment was introduced so that the interpolation works effectively (Watanabe et al., 2005). The peak of the main response of each HRIR was finely adjusted using eight times oversampling, that is, with eight times higher temporal resolution. Because of this time alignment as well as the horizontal HRIR resolution of five degrees, we expected that our VAD had sufficiently good precision. The HRIRs were always updated by reflecting the listeners’ head rotations (3DOF of yaw, pitch, and roll) observed using a motion sensor at the rate of 120 Hz (Fastrak, Polhemus) so that the virtual sound sources can be presented stably at specified positions in terms of the virtual world coordinates. The output signals were presented via headphones (SR202, STAX Ltd.). The total system latency of the VAD used was 12.0 ± 0.05 ms (Yairi et al., 2007, 2008), which is the sum of the latencies of head-tracking, position data transmission, interpolation of head-position, and delay by sound buffer (128 samples). This total system latency value is much less than the detection threshold (DT) of the delay of head-tracking in VADs, which is 50 to 75 ms (Yairi et al., 2007, 2008). Therefore, listeners can localize sound images with no incongruity with their own motion.

In this experiment, the stationary condition and head movement conditions were used. In the stationary condition, listeners listened to the stimulus without moving the head. In a head movement condition, listeners listened to the stimulus while horizontally rotating the head. The sound stimulus in both conditions was pink noise (frequency range, 40 Hz–21.8 kHz; sampling frequency, 48 kHz) convolved with the observer’s HRIR of specified direction. The sound was presented using the VAD. The sound pressure levels of the stimuli were measured using an artificial ear (4153; Bruel and Kjaer) to set it to 65 dB. Both conditions comprised experimental stimulus of two types. These were a still virtual sound source and a moving virtual sound source. The sound source was virtually placed on a circle of 150 cm radius on the horizontal plane centered at the listener’s head. When a still virtual sound source was presented, it was presented at 60° to the right or left of the listener and did not move. In contrast, when a moving virtual sound source was presented, a virtual sound source was first presented at 60° right or left of the listener’s orientation and moved, later in the middle of the presentation, only once on the circle with constant angular velocity from 60° to a more lateral angle with a specified deviation. It was subsequently returned to the original position. These moving processes took 500 ms. In the experiment, a trial consisted of two intervals. In an interval, the still virtual sound source was presented. In the other interval, the moving virtual sound source was presented. The order of these two intervals was randomized in each trial. Listeners were asked to judge in which interval in a trial the sound stimuli moved (two-interval two-alternative forced-choice task, chance rate = 50%). The duration of each of the virtual sound sources was 6 s. After the presentation of the first stimulus, the listener pushed a key to start the second stimulus in 1 s.

Under stationary head conditions, a listener faced forward with the head front 0° and then the two stimuli were presented as described earlier. When a moving sound source was presented, the sound source started moving 2 to 3 s after the start of the presentation; the elapsed time length was selected randomly for each stimulus. The deviation of the moving sound source, which was incremental in terms of absolute values, was chosen randomly from 1°, 2°, 4°, 6°, 8°, 10°, and 12°. This stationary condition comprised four sessions and 280 trials in total (7 sound image movement angles × 2 directions × 10 repetitions). The stimuli were presented with fully randomized order.

Under the head movement condition, a listener faced forward with the head front 0°; then the two stimuli were presented. As soon as each virtual sound source was presented from 60° right or left, listeners were asked to orient toward the sound image in front in 1 s. In other words, the head rotation velocity was 60°/s. A 440 Hz pure tone (duration, 100 ms) was presented three times at 1 s intervals 1 s after sound stimulation presentation to assist observers because it is difficult to control head rotation velocity by instruction alone. Listeners were asked to start head rotation upon completion of the third pure tone presentation. We expected that users would use this step to learn this target rotation velocity. After each stimulus presentation, the average head speed during one head motion was fed back to the listener. In this condition, the virtual sound source started moving when the listener turned his head 30°. Although the manner of the virtual sound source movement was identical to that of the stationary condition, the angular increment was one of 5°, 10°, 20°, 30°, 40°, 50°, and 60°. The session structure was identical to the stationary sound condition. Listeners performed the head movement condition after the stationary condition.

### Results and Discussion

**Figure 1.**
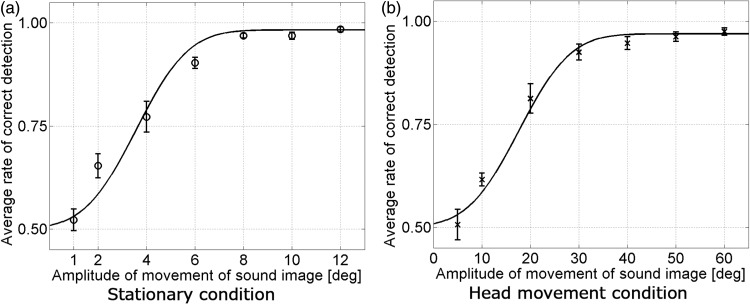
Average rate of correct detection in Experiment 1. Error bars represent the standard deviations.

Results are presented in Figure 1. The average head rotation velocity was 63.5°/s (*SD* = 3.52°/s). We calculated and analyzed the DTs of sound source movement (correct rate of 75%) for each listener based on maximum likelihood fitting to the psychometric functions (0.5 × cumulative normal distribution function + 0.5). Statistical analyses demonstrated that the DT in the head movement condition (17.7°) is significantly greater than that in the stationary condition (3.6°), *t*(7) = 9.93, *p* < .01. Therefore, our results showed that during head rotation, listeners were less sensitive to sound image movement than in the head stationary state. Previous reports have described that if a sound is presented during head movement, the sound localization accuracy is reduced (Brimijoin and Akeroyd, 2014; Cooper et al., 2008; Masumi, Honda, et al., 2014; Masumi, Suzuki, et al., 2014). Our results indicate that the DT of a moving sound image increases during head rotation, as found in the previous studies. However, this experiment involves the following issues: First, the angular amount of virtual sound source movement differed between two conditions. Second, the order effect of head movement was not controlled. Third, only one head rotation velocity was examined. To resolve these issues, we conducted Experiment 2.

## Experiment 2

Listeners were nine male students with normal hearing ability (average age: 22.8 years old). Six of them also participated in Experiment 1. The apparatus and stimuli were identical to those used in Experiment 1.

Experiment 2 consisted of the stationary condition and two head movement conditions (30°/s and 60°/s). When the head rotation velocity was 30°/s, the listener was asked to orient in the direction of the sound image in front in 2 s and to assist observers in maintaining the head rotation velocity. An auxiliary tone similar to that used in Experiment 1 was presented as follows: A 440 Hz pure tone (100 ms) was presented three times at 2 s intervals. Then the listener was asked to start rotating the head after the third presentation of a pure tone. The sound stimulus duration was 10 s. When the velocity was 60°/s, similarly to Experiment 1, a pure tone was presented three times at 1 s intervals. The duration was 6 s. After each stimulus presentation, the average head speed during one head motion was fed back to the listener. Each condition was constructed using three levels of the sound image movement angular amount (5°, 10°, 20°) and two levels of sound image position (60° in right and left). The head movement condition (head stationary, head rotation of 30°/s, and 60°/s) was counterbalanced across listeners. The experiment was performed in three sessions (stationary, 30°/s and 60°/s sessions). The number of trials in each session was 60 in all (3 sound image movement angles × 2 directions × 10 repetitions). The sound image movement angular amount and sound image position were fully randomized.

### Results and Discussion

**Figure 2.**
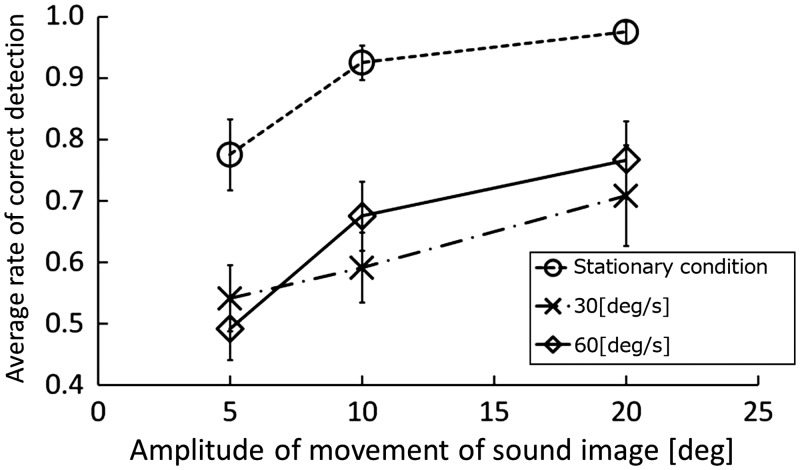
Average rate of correct detection in Experiment 2. Error bars represent the standard deviations.

Results are presented in Figure 2. Average head rotation velocities for 30°/s and 60°/s conditions were, respectively, 33.0°/s (*SD* = 6.20°/s) and 62.9°/s (*SD* = 6.99°/s). A three-way within-subject analysis of variance (ANOVA) of the correct detection rate (DR) was performed, considering the sound image movement angular amount (5°, 10°, 20°), the sound image position (60° in right and left), and the head movement condition (head stationary, head rotation of 30°/s, 60°/s) as factors. Results revealed that main effects of the angular amount, *F*(2, 16) = 31.73, *p* < .001, and of the head movement, *F*(2, 16) = 22.93, *p* < .001, were significant. The other main effect and the interaction were not significant. We conducted post hoc analyses using Ryan’s method to elucidate the main effects. Regarding the angular amount, at 20°, it was detected most easily (DR = 0.87). At 10° (DR = 0.77) and 5° (DR = 0.64), it was detectable but less easily in this order (*ps* < .05). Regarding the head movement, the DR of sound source movement under the head stationary condition (DR = 0.91) was significantly higher than under conditions of head rotation of 30°/s (DR = 0.67) and 60°/s (DR = 0.71) (*ps* < .05). In contrast, no significant difference was observed between head rotation speeds of 30°/s and 60°/s (*p* = .28). Therefore, results demonstrated that similar to Experiment 1 and in agreement with previous studies, the detection of sound source movement is more difficult under head rotation than without head movement. Results demonstrated that this is true not only at the rotation velocity of 60°/s but also at 30°/s.

## General Discussion

Sound image movement detection during horizontal head rotation was investigated using the VAD. Previous reports of some studies have described that when a sound is presented during head movement, the sound localization accuracy is reduced (Brimijoin and Akeroyd, 2014; Cooper et al., 2008; Leung et al., 2008; Masumi, Honda, et al., 2014; Masumi, Suzuki, et al., 2014). Results of Experiment 1 suggest that the DT of a moving sound source during head rotation is greater than that under the stationary condition. Experiment 2 revealed that, in addition to the replication of the results of Experiment 1, the same phenomena occur even with head rotation movement of 30°/s. Our results indicate that an increase in the DRs of sound source movement during head movement is observed in both head velocity conditions.

Previous reports of studies showing that sound image localization accuracy for that sound is reduced fall into two categories. Some studies (Cooper et al., 2008; Leung et al., 2008) have revealed that the phenomenon occurs when the head rotation movement is made rapidly. For instance, Leung et al. (2008) reported that observers’ average head turn speed had mean velocity of 256°/s. Moreover, Cooper et al. (2008), from findings based on data of participants’ head turn velocity, found a mean of 124°/s. Results of other studies suggest little velocity dependence (Masumi, Honda, et al., 2014; Masumi, Suzuki, et al., 2014). For instance, Masumi, Honda, et al. (2014) reported that sound localization accuracy is independent of listeners’ passive head turn speeds of 5 to 20°/s. In this study, the observers’ average head rotation velocities were 63.5°/s for Experiment 1. The average head rotation velocities for 30°/s and 60°/s conditions for Experiment 2 were 33.0°/s and 62.9°/s, respectively. Therefore, results of this study correspond to those of studies that suggest little velocity dependence (Masumi, Honda, et al., 2014; Masumi, Suzuki, et al., 2014). More specifically, our results indicate that deterioration of sound localization accuracy is commonly observed not only from rapid head movement but also from moderate or slow head movement.

Moreover, our results can be regarded as consistent with results reported by Brimijoin and Akeroyd (2014), which were also obtained using VAD. Among the four conditions examined, two were comparable to ours. They are Condition 1, in which both the observer’s head and sound sources were stationary, and Condition 4, in which observers rotated the head several times and the virtual sound sources were moved properly in the egocentric coordinate so that the virtual sound source positions are presented as stationary in the virtual world coordinate. That is, their minimum audible angle (MAA) obtained in their Condition 1 might be compared with our DT obtained in the stationary condition if effects of the unconscious micro head movements could be disregarded. Moreover, their MAA obtained in their Condition 4 might be compared with our DT obtained in the head movement conditions, although the experimental conditions differ somewhat. They presented male and female voices at fixed but different positions in the virtual world coordinate, whereas we presented, in our head movement condition, a pink noise burst initially at 60° in the virtual world coordinate. It was then moved away to a more lateral angle from 60° and back to 60° with a specified directional deviation of 1° to 12°. They reported that the MAA, which they call moving MAA (MMAA), is larger when the head is moving under the proper control of the virtual sound source being fixed in virtual world coordinate (ca. 6°, Condition 4) than when the head and the virtual sound source positions are stationary (ca. 3°, Condition 1). In summary, their results and ours both indicate that the sound localization resolution becomes larger (worse) when observer (listener) is rotating the head. Brimijoin and Akeroyd (2014) also report that MMAA, which shows auditory resolution for static relative positions between sound sources, is larger when the head is moving than when it is stationary, irrespective of whether the virtual sound sources are stationary or moving in the virtual world coordinate. In addition, our results newly indicate that the detection of sound source motion in virtual world coordinate worsens when the head is rotating.

How can localization accuracy deterioration during a listener’s head movement be explained? We attempt to explain this issue based on two models. The first applies a basic cognitive information processing model. In general, in information processing, a tradeoff relation prevails between speed and accuracy (Wickelgren, 1977). On the basis of this model, we have anticipated that the DR of sound source movement under the head rotation of 30°/s was significantly higher than those with head rotation of 60°/s. However, no significant difference was observed between head rotation of 30°/s and 60°/s. Then, the velocity dependence of the phenomena observed in this study is weak. Therefore, an explanation based solely on this model seems inappropriate. The second is a perceptual information processing model. For instance, Viemeister and Wakefield (1991) proposed an auditory information processing model known as the multiple-look model. According to this model, one can expect that sound localization performance worsens with head movements because extra sources of error arise due to the dynamic change of auditory input, rather than simply more information to be integrated. Results of this study showed the expected tendency in that regard. In the multiple-look model, when the sound space perception was examined with this model, auditory input information was first divided into time windows of several millisecond units. Then localization was performed for each divided window. For input sound information, the sound image position was estimated in several-millisecond units. Their outputs were finally integrated to conclude sound image localization. For the head movement conditions examined in this study, in addition to dynamic changes of the information input to the ears because of sound source movement, the information obtained using the listener’s head movement should be integrated with that information. Therefore, under a head movement condition, the information amount to be integrated is substantially greater, in terms of both amount and complexity, than that under a head-stationary condition. Accordingly, the multiple-look model can qualitatively explain the experimentally obtained results that sound localization resolution is worsened by head movements.

As future research directions intended to enrich our understanding, it is important to conduct experiments under actual environments. Furthermore, future studies must investigate sound image movement DTs during faster and slower horizontal head rotation.
